# The Effect of Acute Psychotic Period on Physiological Values Measured in Blood in Male Patients With Schizophrenia

**DOI:** 10.31083/AP53389

**Published:** 2026-08-21

**Authors:** Guler Celikel, Suat Yalcin, Tevfik Kalelioglu, Nesrin Karamustafalioglu

**Affiliations:** ^1^Department of Psychiatry, University of Erzincan Binali Yıldırım, Mengucek Gazi Education and Research Hospital, 24100 Erzincan, Turkey; ^2^Department of Psychiatry, Basaksehir Cam and Sakura City Hospital, 34490 İstanbul, Turkey; ^3^Department of Psychiatry & Neurobehavioral Sciences, University of Virginia School of Medicine, Charlottesville, VA 22908, USA; ^4^Department of Psychiatry, Bakirkoy Prof Mazhar Osman Training and Research Hospital for Psychiatry, Neurology and Neurosurgery, 34140 İstanbul, Turkey

**Keywords:** schizophrenia, osmolarity, blood viscosity, cardiovascular disease, hemodynamics

## Abstract

**Background:**

There is increasing evidence that plasma osmolarity is an independent prognostic factor associated with mortality in patients with cardiovascular disease. Whole blood viscosity (WBV) is also associated with established cardiometabolic risk factors for cardiovascular disease. The present study aimed to investigate the effects of the acute phase of schizophrenia on physiological parameters, including WBV and plasma osmolarity, in male patients with schizophrenia.

**Methods:**

A total of 121 male patients with schizophrenia were included in this study. Routine blood test results were obtained from hospital records, and the laboratory values required for the calculation of viscosity and osmolarity were recorded. Laboratory data from the patients’ most recent acute exacerbation were retrieved from hospital records and used as the acute-phase data.

**Results:**

There was no statistically significant difference in serum osmolarity between the acute and remission phases (*p* = 0.360). Blood urea nitrogen (BUN) and glucose levels were significantly higher during the remission phase, whereas sodium levels were significantly higher during the acute phase. No significant differences were observed in WBV values between the acute and remission phases (*p* > 0.05). Furthermore, there were no significant differences in WBV-related parameters, including hematocrit (HCT) and total protein, between the two phases. Low-density lipoprotein (LDL), high-density lipoprotein (HDL), total cholesterol, and triglyceride levels were significantly higher during the remission phase.

**Conclusions:**

Although differences were observed in osmolarity-related parameters and viscosity-associated metabolic variables between the acute and remission phases in male patients with schizophrenia, no statistically significant differences were found in serum osmolarity or WBV between the two phases.

## Main Points

1. Serum osmolarity and whole blood viscosity (WBV) values measured during acute and remission periods did not significantly differ in male patients with schizophrenia.

2. Despite changes in metabolic parameters including glucose, sodium, and blood urea nitrogen (BUN), osmotic and hemodynamic balance appeared to be preserved across clinical phases.

3. No significant association was observed between medication type and percentage changes in serum osmolarity or whole blood viscosity values.

## 1. Introduction

Schizophrenia is a serious mental illness, in which the risk of mortality is two to four times higher than that in the general population [1,2]. Like the general population, the most common cause of death in patients with schizophrenia in recent years is cardiovascular diseases (CVD) [3,4]. Smoking, diabetes mellitus (DM), hypertension (HT), use of antipsychotics, hyperlipidemia, sedentary lifestyle and metabolic syndrome (MS) are the most common risk factors of CVD in patients with schizophrenia [2,5]. In recent years, the increased use of atypical antipsychotics in schizophrenia treatment has caused serious metabolic side effects such as weight gain, hyperlipidemia, pancreatitis, diabetes and impaired glucose tolerance and an increase in CVD risk and mortality [6,7,8].

The role of dopamine and its receptors in the central nervous system has been extensively studied, and its effects on peripheral tissues such as the eye, cardiovascular system, pancreas and renal system have been demonstrated in studies [9,10]. As an endogenous modulator, dopamine acts as a local natriuretic system that regulates kidney functions such as fluid electrolyte balance, blood pressure regulation, and renal redox homeostasis [11]. Renal dopamine is produced in the kidneys, independent of neuronal dopamine synthesis [12]. The major regulatory systems that provide sodium homeostasis are the renal dopaminergic system and the renin-angiotensin system [13].

Osmolarity is associated with mortality in various diseases, especially cardiovascular diseases, and it has been predicted that it can be used as a prognostic factor to detect populations at high risk [14,15,16]. In another study, it was determined that osmolarity in patients with ST-elevation myocardial infarction (STEMI), and that osmolarity may be useful in predicting a high-risk group for STEMI [15].

In light of these findings, and considering the pathophysiology of schizophrenia and the mechanisms of action of antipsychotic medications, it was hypothesized that dopamine hyperactivity during the acute phase of schizophrenia could affect natriuresis and water permeability through the renal dopaminergic system. Furthermore, treatment with dopamine antagonist antipsychotics during remission may alter water and electrolyte balance, and serum osmolarity may therefore serve as a predictive marker of cardiovascular risk in patients with schizophrenia. However, there are insufficient data in the literature regarding the relationship between osmolarity and psychiatric disorders.

Cardiovascular disease risk factors such as hypertension, diabetes, smoking, low-density lipoprotein (LDL) cholesterol increase and age are associated with whole blood viscosity (WBV) increase, therefore it was stated that rheological changes could predict ischemic vascular diseases. It has been proven in clinical studies that WBV is the causal determinant of atherosclerosis [17].

As indicated in several studies, viscosity is associated with cardiovascular disease risk and total mortality [18,19,20,21].

Considering the established associations of serum osmolarity and WBV with cardiovascular risk and mortality, it remains unclear whether these parameters differ between the acute and remission phases of schizophrenia. Therefore, the present study aimed to compare serum osmolarity, WBV, and their related physiological parameters between the acute and remission phases in male patients with schizophrenia.

## 2. Materials and Methods

### 2.1 Participants

In the present study, 1020 patients who were being followed up at the outpatient clinic of Bakirkoy Prof Mazhar Osman Training and Research Hospital for Psychiatry were consecutively recruited between April and July 2019. Patients diagnosed with schizophrenia who had been hospitalized at least once more than six months previously and were in remission were included in the study.

Of the 1020 patients, 899 were excluded from the study for the following reasons: 195 had a diagnosis of bipolar disorder, 6 had delusional disorder, 33 had schizoaffective disorder, 6 had organic mental disorder, 285 had no history of acute hospitalization, 75 had been hospitalized within the previous six months, 31 had a comorbid psychiatric disorder, 9 had alcohol use disorder, 67 had substance use disorder, 147 had medical comorbidities, 14 did not have laboratory data from their acute hospitalization, 4 had a history of hyponatremia, 10 had polydipsia, 10 were receiving carbamazepine treatment, and 7 were older than 65 years.

The remaining 121 male patients, aged 18–65 years, who were diagnosed with schizophrenia according to the Diagnostic and Statistical Manual of Mental Disorders, Fifth Edition (DSM-5) criteria, were in remission, and agreed to participate in the study were included. The Clinical Global Impression (CGI) scale and the Positive and Negative Syndrome Scale (PANSS) were used to determine whether the patients diagnosed with schizophrenia were in remission (CGI severity score < 5 and no hospitalization within the previous six months). Review of the patients’ most recent hospitalization records confirmed that they had been in an acute episode.

The results of the routine blood tests performed during the first days of hospitalization and at follow-up were obtained from the hospital records, and the laboratory values required for the calculation of viscosity and osmolarity were recorded.

To exclude patients with comorbid medical conditions, eligibility was assessed according to current diagnostic and treatment guidelines, and only patients whose laboratory values were below the treatment thresholds were included in the study. Accordingly, a fasting blood glucose level ≥126 mg/dL was used as the diagnostic threshold for diabetes mellitus, whereas LDL cholesterol ≥190 mg/dL and triglyceride (TG) levels ≥500 mg/dL were used as treatment thresholds for hyperlipidemia in patients without additional cardiovascular disease risk [22,23].

WBV is evaluated at low and high shear rates with the Simone formula as:

At low shear rate (LSR) WBV (0.5 sec^–1^) = (1.89 × hematocrit [HCT]) + 3.76 total protein [TP] (g/dL) – 78.42;

High shear rate (HSR) WBV (208 sec^–1^) = (0.12 × HCT) + 0.17 TP (g/dL) – 2.07 [24,25,26].

Serum osmolarity is maintained at 275–295 mOsm/L H_2_O normally and calculated from Sodium (Na^+^), glucose and blood urea nitrogen (BUN) according to the formula (2 × Na^+^) + (BUN / 2.8) + (Glucose / 18) [27].

### 2.2 Statistical Analysis

Statistical analyses were performed using IBM SPSS Statistics version 21.0 (IBM Corp., Armonk, NY, USA). Continuous variables were presented as minimum–maximum values, mean ± standard deviation (SD), and median values, whereas categorical variables were presented as frequencies and percentages. The normality of the continuous variables was assessed using the Kolmogorov–Smirnov test. Comparisons between independent groups were performed using the independent-samples *t*-test, Mann–Whitney U test, Kruskal–Wallis test, and one-way analysis of variance (ANOVA), as appropriate. For within-group comparisons, paired-samples *t*-tests were used for normally distributed variables, whereas Wilcoxon signed-rank tests were used for non-normally distributed variables. A *p*-value < 0.05 was considered statistically significant.

## 3. Results

The mean age of the patients was 38.05 ± 10.28 years. The mean duration of illness of the patients participating in the study was 14.94 ± 8.74 years and their mean age at first treatment was 23.02 ± 6.72 years. The mean number of hospitalizations of the patients was 3.58 ± 3.42, and the mean duration of their hospitalizations was 18.96 ± 9.99 days. Antipsychotic medication used was as follows: 4.13% (n = 5) were typical antipsychotics, 37.19% (n = 45) atypical antipsychotics, 14.05% (n = 17) clozapine, and 44.63% (n = 54) medication in combination (Table 1).

**Table 1.** **Demographic and clinical properties of the patients**.

	Min–Max	Mean ± SD
Age	20–65	38.05 ± 10.28
Duration of illness (years)	1–42	14.94 ± 8.74
Age of first treatment	14–48	23.02 ± 6.72
Number of hospitalizations	1–20	3.58 ± 3.42
Duration of hospitalization (day)	1–64	18.96 ± 9.99
	**n**	**%**
Typical antipsychotics	5	4.13
Atypical antipsychotics	45	37.19
Clozapine	17	14.05
Combination therapy	54	44.63

SD, standard deviation.

The mean serum osmolarity level of the patients was 296.83 ± 6.15 mOsm/L in the acute period, and 296.13 ± 6.35 mOsm/L in the remission period. There was no statistically significant difference between acute and remission periods with regard to the serum osmolarity levels (*p* = 0.360).

The components of serum osmolarity were as follows: median glucose (85 mg/dL in acute phase, 95 mg/dL in remission), and median BUN (23.57 mg/dL in acute period, 25.43 mg/dL in remission) levels were statistically significantly higher in the remission period than were those in the acute period (*p* < 0.05). The median sodium level was statistically significantly higher in the acute period (142 mmol/L) than was that in the remission period (140.5 mmol/L) (*p* < 0.05) (Table 2).

**Table 2.** **Comparison of serum osmolarity and parameters in acute and remission periods of schizophrenia**.

	Acute (n = 121) Mean ± SD	Remission (n = 121) Mean ± SD	*p*
Serum osmolarity^α^ (mOsm/L)	296.83 ± 6.15 *	296.13 ± 6.35 *	0.360
	**Min–Max (Median)**	**Min–Max (Median)**	
Glucose^β^ (mg/dL)	60–112 (85.00) †	72–125 (95.00) †	**<0.001**
Na^β^ (mmol/L)	133–146 (142.00) †	133–147 (140.50) †	**0.005**
BUN^β^ (mg/dL)	9–53 (23.57) †	10–50.52 (25.43) †	**0.006**

^α^ Paired Sample *T* Test. ^β^ Wilcoxon Signed Rank Test. * Data are presented as mean ± SD. † Data are presented as Minimum–Maximum (Median). The bolded values indicate statistically significant results.

Regarding WBV, the mean LSR was 53.80 ± 23.14 in the acute period and 51.88 ± 16.87 in the remission period; the mean HSR was 16.95 ± 1.13 in the acute period and 16.87 ± 0.79 in the remission period. There was no statistically significant difference between the serum viscosity values as HSR and LSR during the acute and remission periods of the patients (*p* = 0.472 for HSR; *p* = 0.428 for LSR). There was no statistically significant difference between acute and remission periods for components of WBV as; total protein (acute phase 7.03 ± 0.52 g/dL, remission period 6.98 ± 0.42 g/dL) and HCT (acute phase 30.80–53.90 (45.3), remission 35.30–50.10 (45.3)) (*p* = 0.303 and *p* = 0.782, respectively) (Table 3).

**Table 3.** **Comparison of WBV and parameters in acute and remission periods of schizophrenia**.

	Acute (n = 121) Mean ± SD	Remission (n = 121) Mean ± SD	*p*
WBV at LSR^α^ (cP)	53.80 ± 23.14 *	51.88 ± 16.87 *	0.428
WBV at HSR^α^ (cP)	16.95 ± 1.13 *	16.87 ± 0.79 *	0.472
Total protein^α^ (g/dL)	7.03 ± 0.52 *	6.98 ± 0.42 *	0.303
	**Min–Max (Median)**	**Min–Max (Median)**	
Hematocrit^β^ (%)	30.80–53.90 (45.30) †	35.30–50.10 (45.30) †	0.782

^α^ Paired Sample *T* Test. ^β^ Wilcoxon Signed Rank Test. * Data are presented as mean ± SD. † Data are presented as Minimum–Maximum (Median). WBV, whole blood viscosity; LSR, low shear rate; HSR, high shear rate.

No statistically significant differences were determined in the percentage changes in patients’ serum osmolarity and whole blood viscosity values according to medication type (*p* > 0.05) (Table 4).

**Table 4.** **Comparison of percentage changes in osmolarity and viscosity parameters according to antipsychotic medication class**.

Change between two measurements (%)	Typical AP Mean ± SD	Atypical AP Mean ± SD	Clozapine Mean ± SD	Combination Mean ± SD	F/Chi-square	*p*
Osmolarity^α^	–0.555 ± 1.53	–0.219 ± 2.80	–0.067 ± 3.40	–0.189 ± 2.61	0.041	0.989
HSR^α^	–1.491 ± 4.04	0.922 ± 7.54	–1.73 ± 6.56	–0.30 ± 7.50	0.572	0.635
	**Min–Max (Median)**	**Min–Max (Median)**	**Min–Max (Median)**	**Min–Max (Median)**		
LSR^β^	–47.4–37.9 (–10.440)	–60.2–2652.7 (7.43)	–56.4–110.8 (–16.95)	–70.4–1219.2 (–2.61)	1.029	0.794

^α^ One-way analysis of variance (ANOVA) was applied. ^β^ Kruskal–Wallis test was applied; df = 3. AP, antipsychotic.

No statistically significant correlations were observed between clinical variables, including age, the time interval between laboratory measurements, and duration of illness, and the percentage changes in LSR, HSR, or serum osmolarity values (all *p* > 0.05).

No statistically significant correlations were observed between the percentage changes in serum osmolarity and the percentage changes in blood lipid parameters (all *p* > 0.05).

Analysis of the linear relationship between percentage changes in WBV and blood lipid parameters revealed statistically significant weak positive correlations between percentage changes in HSR and percentage changes in LDL, high-density lipoprotein (HDL), and triglyceride levels (all *p* < 0.05). In addition, a statistically significant moderate positive correlation was observed between percentage changes in HSR and total cholesterol levels (*p* < 0.001).

For LSR, percentage changes in WBV showed statistically significant weak positive correlations with percentage changes in LDL and triglyceride levels (all *p* < 0.05). However, no statistically significant correlation was observed between percentage changes in LSR and HDL levels (*p* > 0.05). Furthermore, a statistically significant moderate positive correlation was identified between percentage changes in LSR and total cholesterol levels (*p* < 0.001) (Table 5).

**Table 5.** **Correlation analysis between percentage changes in osmolarity/viscosity parameters and clinical variables**.

	% Osmolarity	% HSR	% LSR
Age	r = 0.061 *p* = 0.518	r = 0.070 *p* = 0.474	r = 0.071 *p* = 0.465
Time interval between laboratory measurements	r = 0.139 *p* = 0.138	r = 0.063 *p* = 0.517	r = 0.054 *p* = 0.581
Duration of illness	r = 0.086 *p* = 0.359	r = 0.044 *p* = 0.654	r = 0.045 *p* = 0.649
LDL	r = 0.097 *p* = 0.373	r = 0.251 *p* = 0.021*	r = 0.277 *p* = 0.011*
Total cholesterol	r = 0.134 *p* = 0.189	r = 0.375 *p* < 0.001**	r = 0.393 *p* < 0.001**
HDL	r = –0.028 *p* = 0.785	r = 0.217 *p* = 0.037*	r = 0.180 *p* = 0.084
Triglyceride	r = 0.196 *p* = 0.054	r = 0.269 *p* = 0.009*	r = 0.285 *p* = 0.005*

% Change between Two Measurements. Spearman correlation analysis was applied. *p < 0.05 and **p < 0.001 were considered statistically significant. The bolded values indicate statistically significant results. LDL, low-density lipoprotein; HDL, high-density lipoprotein.

## 4. Discussion

To our knowledge, this is the first study to compare serum osmolarity and WBV between the acute and remission phases of schizophrenia in male patients. The main findings of this study were that serum osmolarity and WBV did not differ significantly between the acute and remission phases despite significant changes in glucose, sodium, BUN, and lipid parameters. Although glucose and BUN levels were significantly higher during the remission phase and sodium levels were significantly higher during the acute phase, no statistically significant difference was observed in serum osmolarity. Additionally, no significant differences were observed in WBV-related parameters, including hematocrit and total protein. Furthermore, no significant associations were found between changes in serum osmolarity or WBV and clinical variables, including medication type, age, duration of illness, and the number of hospitalizations. However, changes in lipid parameters were significantly correlated with changes in WBV, whereas no such association was observed for serum osmolarity.

It has been reported that the life expectancy of patients with schizophrenia is reduced by 10–20% compared with that of the general population and that the leading cause of mortality is CVD [1,3,28]. Several studies have suggested that serum osmolarity may be used as a prognostic marker for identifying high-risk patients and predicting all-cause mortality in cardiovascular diseases such as myocardial infarction and heart failure [15,16,29].

In a study conducted by Gunduz-Bruce et al. [30], sodium levels were reported to be higher in patients with schizophrenia than in healthy controls, whereas plasma osmolarity was also elevated but did not reach statistical significance. In addition, the association between osmolarity and brain ventricle dimensions observed in healthy controls was not detected in patients with schizophrenia, which was interpreted as a possible disturbance in cerebral fluid regulation mechanisms, even in the absence of symptoms such as polydipsia in first-episode schizophrenia [30]. In the present study, although the acute and remission phases of schizophrenia were compared without including a healthy control group, similar results were obtained. These findings suggest that osmoregulation is maintained and hemodynamic balance is preserved despite increases in cardiometabolic risk factors, such as elevated glucose levels following treatment. Furthermore, the reported effectiveness of antipsychotics such as clozapine in the treatment of polydipsia may suggest that disease-related alterations in fluid regulation mechanisms are modified during treatment or compensated for by mechanisms involved in sodium homeostasis [31].

In their study of 274 male patients with schizophrenia in remission without a healthy control group (2025), Özönder Ünal et al. [32] reported that serum osmolarity values remained within the normal physiological range but were close to its upper limit. The authors interpreted this finding as indicating subtle yet potentially clinically relevant alterations in fluid balance that may persist even during the remission phase of schizophrenia. Similarly, in the present study, although no significant difference was observed in serum osmolarity between the acute and remission phases, serum osmolarity values in both phases were close to or slightly above the upper limit of the physiological range. These findings may suggest that, despite preserved osmotic balance across clinical phases, subtle alterations in fluid regulation mechanisms may persist in patients with schizophrenia [32].

In their study, which included patients with schizophrenia, Kalelioğlu et al. [33] compared episodes of neuroleptic malignant syndrome (NMS) with non-NMS hospitalizations of the same patients, which served as the control group, and demonstrated that serum osmolarity was significantly higher during NMS episodes than during non-NMS hospitalizations. These findings suggest that increased osmolarity may represent a marker of relative hemoconcentration in NMS. Considering that autonomic instability is one of the diagnostic criteria for NMS, increased osmolarity in the NMS group may be an expected finding. Similarly, in the present study, serum osmolarity values measured during the acute and remission phases were compared within the same patient group. Despite changes in cardiometabolic risk factors associated with antipsychotic treatment, the absence of a significant difference in serum osmolarity between the two phases may suggest that osmoregulation is preserved across the clinical phases of schizophrenia [33].

The role of renal dopamine in sodium homeostasis, together with evidence suggesting that dopamine receptor dysfunction contributes to the pathogenesis of hypertension, raises the possibility that dopamine antagonists used in the treatment of schizophrenia may influence serum osmolarity. However, the absence of a difference in serum osmolarity between the acute and remission phases may be related to the predominant role of D1 and D3 receptors in sodium homeostasis, whereas the effects of antipsychotics are primarily mediated through D2 receptor antagonism [8]. Previous studies have also demonstrated that antipsychotics may increase aldosterone and arginine vasopressin (AVP) secretion through D2 receptor antagonism [34,35]. Overall, sodium and water metabolism are regulated by multiple interacting mechanisms. Although it is difficult to evaluate overall osmoregulation based on individually examined pathways, the present findings may suggest that compensatory mechanisms become activated during both the acute and remission phases of schizophrenia, thereby helping to maintain hemodynamic balance despite disease-related changes and the effects and side effects of antipsychotic treatment. Further studies are needed to clarify the underlying mechanisms and their contribution to osmoregulation in schizophrenia.

Walsh et al. [34] reported that, in male patients with first-episode schizophrenia who were not receiving medication, basal cortisol and adrenocorticotropic hormone (ACTH) levels were significantly higher than those in healthy controls, whereas basal AVP levels were lower, although the latter difference did not reach statistical significance. The elevated basal ACTH and cortisol levels were considered to support the hypothesis of increased hypothalamic–pituitary–adrenal (HPA) axis activity in schizophrenia and may be explained by dopaminergic hyperactivity [34]. In another study, patients with schizophrenia had lower plasma osmolarity and aldosterone levels than healthy controls, whereas AVP and atrial natriuretic peptide (ANP) levels were higher. It has been suggested that long-term antipsychotic treatment or polydipsia may contribute to water and electrolyte imbalance in patients with chronic schizophrenia. However, Kudoh et al. [35] did not report whether the patients included in their study had polydipsia.

Several studies have reported differing findings regarding ACTH, cortisol, AVP, and serum osmolarity levels in patients with schizophrenia. These discrepancies may be related to differences in patient selection. Factors such as disease phase (acute or chronic), medication use, the presence of polydipsia, and medication type may all contribute to these differences [34,35]. In the present study, serum osmolarity values measured during the acute and remission phases did not differ significantly. The exclusion of patients with polydipsia may suggest that the balance between the mechanisms involved in osmoregulation remains preserved and that hemodynamic stability is maintained.

WBV has been associated with cardiovascular disease risk and overall mortality and correlates with major cardiovascular risk factors, including obesity, dyslipidemia, hypertension, and diabetes mellitus. Several studies have suggested that WBV is a causal determinant of atherosclerosis and that rheological changes may be useful in predicting ischemic vascular disease [17,18,36].

In a naturalistic retrospective study conducted in Poland, red blood cell parameters were compared among patients with acute schizophrenia, bipolar disorder, and depression. Mean red blood cell (RBC), hemoglobin (HGB), HCT, and mean corpuscular hemoglobin concentration (MCHC) values were significantly higher in patients with schizophrenia than in the other patient groups. It has been suggested that decreased erythrocyte deformability and increased erythrocyte aggregation in patients with schizophrenia are associated with an increased risk of cardiovascular disease [37].

In a study conducted by Balcıoğlu et al. [38], whole blood viscosity values were lower in both first-episode patients and patients with acute exacerbation of schizophrenia than in healthy controls; however, no significant difference was observed between the two patient groups. In another study by the same authors, patients with treatment-resistant schizophrenia had lower whole blood viscosity values at both low and high shear rates than healthy controls, whereas patients in remission demonstrated reduced viscosity only at high shear rates. However, no significant difference was observed between the treatment-resistant and remission groups in terms of whole blood viscosity [39].

Yao and van Kammen [40] investigated erythrocyte membrane dynamics in patients with schizophrenia and reported that changes in erythrocyte membrane fluidity were independent of antipsychotic treatment, plasma cholesterol levels, and clinical variables, and that decreased membrane fluidity was associated with psychosis.

The main determinants of WBV are hematocrit, erythrocyte deformability, total protein, and plasma viscosity [36]. In the present study, no significant differences in WBV were observed between the acute and remission phases of schizophrenia. Furthermore, the absence of significant differences in whole blood viscosity between the two phases may suggest that disease-related alterations persist across different clinical stages. Consistent with the findings of Yao and van Kammen [40], no association was observed between whole blood viscosity and medication type, depot antipsychotic use, or other clinical variables.

In a review by Kensey [17] examining the relationship between cardiovascular disease and hemorheological variables, WBV was reported to be a causal determinant of atherosclerosis, and increased viscosity was associated with other cardiovascular risk factors. Cardiovascular risk factors, including LDL cholesterol, total cholesterol, HDL cholesterol, and triglyceride levels, were correlated with whole blood viscosity [17]. Consistent with the literature, weak correlations were observed between changes in WBV and changes in LDL, HDL, and triglyceride levels in the present study, whereas a moderate correlation was observed with total cholesterol.

These findings may indicate that serum osmolarity and WBV remain relatively stable despite short-term clinical changes in patients with schizophrenia and may reflect longer-term physiological regulation rather than acute psychiatric status.

### Limitations

Limitations of the present study included several factors. First, the sample size was relatively small. In addition, the patient group consisted of only male patients, so the effects of the menstrual cycle on osmolarity and viscosity, as well as sex differences, were not confounding factors, since previous studies have demonstrated menstrual cycle–related changes in blood viscosity, serum osmolarity, and fluid homeostasis [41,42,43]. Since only male patients were included in the present study, the findings cannot be generalized to female patients with schizophrenia. Future studies including female participants should be conducted to clarify potential sex-related differences and improve the generalizability of the findings. Second, although the reliability of the formulas used to calculate serum osmolarity and WBV has been demonstrated in previous studies, direct measurement of serum osmolarity with an osmometer and WBV with a viscometer would provide more accurate results than indirect calculations using formulas. Third, although blood samples were collected after 8 hours of fasting, differences in diet and exercise habits before blood sampling may have affected the laboratory values. Fourth, the fact that hydration status during the acute phase was not assessed using parameters such as dehydration, dry skin or mucous membranes, and urine specific gravity constitutes a limitation. Fifth, the history of polydipsia was obtained from the patients and their families and, during the acute period, from hospital records. However, since this information was based on subjective evaluation and retrospective data, its reliability should be interpreted with caution. Sixth, another limitation of this study is the heterogeneity of antipsychotic treatments and the inability to standardize medication doses. Considering the known metabolic and hematological effects of different antipsychotic classes, particularly clozapine, their potential impact on serum osmolarity and WBV measurements cannot be excluded. Finally, the retrospective nature of the study and the absence of a healthy control group are other limitations.

## 5. Conclusions

In conclusion, in the present study, the changes in serum osmolarity and WBV were investigated in terms of CVD risk in acute and remission periods of schizophrenia. In the remission period, an increase in glucose and BUN values, which are among the osmolarity parameters, and a decrease in sodium values were detected, however, hemodynamic balance appeared to be preserved and no significant difference was observed in serum osmolarity values. In addition, no significant differences were observed in viscosity parameters such as HCT and total protein or in WBV values determined during the acute and remission periods. In future studies, larger sample sizes and more heterogeneous populations, including female patients and healthy controls should be included to improve the generalizability of these findings and to understand the role of osmolarity and hemorheological changes in schizophrenia better.

## Data Availability

The data that support the findings of the present study are available from the corresponding author upon reasonable request.
